# Host species vary in infection probability, sub-lethal effects, and costs of immune response when exposed to an amphibian parasite

**DOI:** 10.1038/srep10828

**Published:** 2015-05-29

**Authors:** Jon Bielby, Matthew C. Fisher, Frances C. Clare, Gonçalo M. Rosa, Trenton W. J. Garner

**Affiliations:** 1 The Institute of Zoology, The Zoological Society of London, Regent’s Park, London, NW1 4RY, UK; 2 Department of Infectious Disease Epidemiology, Imperial College London W2 1PG, UK; 3 Durrell Institute of Conservation and Ecology, School of Anthropology and Conservation, University of Kent, Canterbury, Kent, CT2 7NR, UK; 4 Centre for Ecology, Evolution and Environmental Changes (CE3C), Faculdade de Ciências da Universidade de Lisboa, Bloco C2, Campo Grande, 1749-016 Lisboa, Portugal

## Abstract

The amphibian parasite *Batrachochytrium dendrobatidis* (*Bd*) is regarded as an extreme generalist, infecting over 500 species, but amongst these hosts there exists a great deal of variation in the susceptibility to and the costs of parasite exposure. We use two infection experiments to determine whether inter-specific variation in the sublethal and lethal effects of parasite exposure exist in two host species. We then tested the relative roles of host density and diversity on infection probability of a focal susceptible host. Our results show significant heterogeneity in host species response to parasite exposure, and that both lethal and sub-lethal costs exist in individuals that are able to resist infection, indicating that successful immune response to infection comes at a cost. Further, we show that increasing host density significantly increased the likelihood of susceptible individuals becoming infected with *Bd* irrespective of host diversity and variation in host susceptibility. These results suggest that populations of resistant species are likely to suffer ill-effects of exposure to *Bd* regardless of their infection status, and that at the stage of initial infection there was no support for the dilution of transmission events, in contrast to other studies that focus on subsequent transmission of infection.

The majority of parasites infect multiple hosts^1^, but they do so to varying degrees; even the most generalist parasites exhibit considerable variation in how frequently and heavily they infect species within their host-base^2^. In some host species many individuals will be infected, and these infections may be severe with a high number of individual parasites per host, whereas in other host species infections will be less common and infections will tend to be lighter. This inter-specific variation in infection frequency and severity is not the only way species respond differently to the same parasite; it is mirrored in other aspects of host-parasite dynamics, such as differences in the consequences of parasite exposure, and the roles different host species play in transmission of infection within a community.

Different host species exhibit a great deal of variation in the consequences of parasite exposure. Hosts may experience significant inter-specific variation in mortality rates and population trajectories as a result of parasite exposure^2^, but variation may be more subtle and still have an effect on individual hosts, their populations and the communities in which they live. The sub-lethal effects of parasite exposure can be considerable at the level of the individual and may scale up to the level of the population, yet they are often overlooked in studies of wildlife disease. Sub-lethal infections can reduce fecundity, increase developmental time and act as a destabilising force in host populations^3^,^4^. Further, negative consequences for the individual do not rely on infection taking place: in a range of taxa, the energetic cost of mounting a successful immune response has been shown to result in reduced growth rates^5^, activity levels^6^, reproductive outputs^7^, body condition^6^, and competitive ability^8^. So, even in the absence of infection there may be important sub-lethal costs related to pathogen exposure, which may translate into population-level outcomes.

Given the observed variation in infection frequency, burden, and consequence, it is no surprise that hosts also contribute unevenly to the transmission of infection within a community^2^. This heterogeneity in transmission of infection results in different host species playing different roles in the persistence and transmission of infection. Examples of different roles that hosts species play fill within a community, include reservoirs, vectors, and amplification and dilution hosts^9^. In the case of the latter, relatively resistant hosts could act to buffer against the spread, severity and effects of parasite infection via the ‘dilution effect’^10^,^11^,^12^. The dilution effect has a number of working definitions^12^, and therefore mechanisms by which it may be observed, but the overall idea is that the dilution effect occurs when an increase in host diversity somehow dilutes the transmission process, thereby reducing the level of disease risk^11^.

*Batrachochytrium dendrobatidis* has been regarded as a host generalist parasite, known to have infected over 500 species^13^. While it has been found on every continent on which amphibians are found^13^, there exists a great deal of variation in the severity and impacts of infection^14^. At the broader taxonomic level recent research focussing on European species highlights how infection levels are geographically consistent for certain widespread and abundant clades^15^. This consistency in infection levels suggests that despite the existence of some intraspecific variation^16^, relative species susceptibility is consistent for a given level of parasite exposure.

In this study we investigated the existence and implications of heterogeneity in response to exposure to *Bd* in two widespread, locally abundant European species, the European common toad, *Bufo bufo*, and the European common frog, *Rana temporaria*. Using these two species we tested three different hypotheses. By implementing an infection challenge experiment, we first tested the hypothesis that, in keeping with the broader-scale, field-based findings of Balaz *et al*^15^, the different focal species have different susceptibility to infection. Using the same experiment, we also tested the hypothesis that, in addition to any observed differences in susceptibility to infection, the two species suffer different costs attributable to parasite exposure. To address the second hypothesis we measured mortality and one sub-lethal cost (change in body mass). Our third hypothesis was that an increased diversity may lead to a dilution effect at the initial infection stage of the transmission process, whereby initial infection probability would be lower within a higher diversity treatment. To test this hypothesis, we aimed to determine whether the density and diversity of available hosts affected the probability of initial infection of a focal host species, *Bufo bufo*, known to be susceptible to infection with, and suffer mortality due to *Bd*^17^,^18^,^19^.

## Methods

For each experiment, we collected ten strings of *Bufo bufo* (hereafter ‘*Bufo*’) spawn and ten clutches of *Rana temporaria* (hereafter ‘*Rana*’) spawn at one breeding site in early March. Hatched tadpoles of each species were mixed and cohoused outdoors as single-species groups in 90 L tubs filled with aged tap water. Tadpoles were fed Tetra TabiMin tablets *ad libitum* until metamorphosis. Animals that completed metamorphosis Gosner stage 46^20^; were transferred to an outdoor enclosure, one per species, and fed *ad libitum* crickets until entry into experiments.

All experimental procedures were done in an indoor facility where room temperature was held at a constant 18 °C with a 12:12 day/night cycle. Experiments were reviewed and approved by ethics committees at the Institute of Zoology and Imperial College London, and were conducted in accordance with Home Office licensing regulations.

### Experiment 1: Individual host species exposures and responses

Metamorphic individuals of both species were allocated to one of three *Bd* dose categories (high, low and sham infection as per Garner *et al*. 2009^18^). Each of the six experimental treatments (two species crossed by three dosing treatments) contained 30 animals, with mass (measured to the nearest 0.01 of a gram) balanced among exposure treatments within species. We exposed each metamorphic animal individually to *Bd* for 5 h in a Petri dish containing 30 mL of aged tap water and the appropriate dose of *Bd*. Dose volume was controlled across exposure treatments (200 μL). We initially determined zoospore (zsp) concentration of stock culture BdGPL IA-42^21^, using a haemocytometer and diluted to the required dose with sterile media. High dose treatment animals were exposed to 16,000 actively swimming zoospores and low dose treatment animals were exposed to 160 actively swimming zoospores (1/100 of the high dose). Negative controls were sham exposed to 200 μL of sterile media.

After exposure each animal was transferred to an individual 700 mL volume plastic box lined with moistened paper towelling and containing a small plastic cover object as a refuge. Animals were housed in this manner for the duration of the experiment (24 days), or until death. Metamorphic individuals were fed *ad libitum* crickets every other day and paper towelling was replaced every four days. During the course of both experiments we monitored food consumption and recorded any loss of appetite. The survival of all animals was tracked for 24 days post-exposure, after which all surviving individuals were weighed to the nearest 0.01 of a gram and euthanized. The experiment was run for 24 days as previous research has shown this to be a sufficient time period for infection to take place and proliferate to a level sufficient for detection^17^,^18^.

To ascertain infection status of all experimental animals and infection burden of infected *Bufo* (see below) we used quantitative real-time PCR^18^,^22^ (see Garner *et al*. 2009 for detailed explanation of molecular diagnostic procedures and standards). Infection status was assessed at the point of death or day 24, whichever came first. From the output of this process we were able to diagnose whether an individual was infected or not and, if infected, the intensity of that infection. The units of infection intensity were mean *Bd* genomic equivalent (GE), one GE being equivalent to the amount of fungal DNA present in a single infectious zoospore.

We used Cox proportional hazard (CPH) models to investigate which factors influenced survival. This analysis was only implemented for *Bufo* because no mortality occurred in *Rana*. The explanatory variables of interest for these analyses were body mass of experimental animals before exposure to *Bd*, whether an individual was exposed to *Bd*, the size of the dose administered, and whether an individual was infected at death or at the end of the experiment. Full models including all variables were reduced to a minimum adequate model with the removal of non-significant terms.

Pre-exposure body mass is known to be an important determinant of the severity of infection in overwintered *Bufo* metamorphs^17^. In order to determine whether *Bufo* body mass before exposure to *Bd* affected infection burden, we used a negative binomial generalised linear model, using the function glm.nb from the R library MASS. We first reduced the *Bufo* dataset to those individuals where infection was detected: this only involved a subset of individuals from the high-dose treatment because this was the only treatment in which infections were detected.

Because other studies have shown that exposure to *Bd* can affect growth^23^,^24^,^25^, we investigated how dose affected the proportion change in body mass for both species separately. To do this we used a one-way analysis of variance, with dose as a factor, and we log-transformed the proportion change in body mass to meet expectations of normality. We used Tukey’s honest significant difference test to determine *post hoc* where differences amongst treatments occurred.

### Experiment 2: Exposing single *Bufo*, cohoused *Bufo* and cohoused *Bufo* and *Rana*

Results from Experiment 1 (see below) and previous studies^17^,^18^,^21^,^26^ showed that *Bufo* are a susceptible host that experiences costs associated with exposure to and infection with *Bd*. Results from Experiment 1 also revealed that *Rana* do not become infected when exposed for 5 hours with up to 16,000 active zoospores. Accordingly, for the purposes of this experiment we defined *Bufo* as a susceptible host species and *Rana* as a resistant host species.

Following the experimental design of Johnson *et al*. 2008^27^, we allocated individuals to one of nine treatments, split into three groups based on the number of amphibians in each replicate (host density) and the number of species in each replicate (host diversity). Replicates either contained a single metamorphic *Bufo* (low density, low diversity), two *Bufo* (high density, low diversity) or one *Bufo* and one *Rana* (high density, high diversity). These three groups were subdivided into three *Bd* dose categories (high, 60,000 zsps; low, 6,000 zsp; sham, 0 zsps, all doses were controlled to a 200 μL volume) with each of the resulting nine treatments replicated 14 times. *Bufo* were randomly allocated to treatment by mass. Animals were exposed to their respective doses in Petri dishes containing 25 mL of aged tap-water as well as the relevant dose of *Bd*. After five hours, all *Bufo* from the single *Bufo* treatment and the *Bufo*/*Rana* treatment, and one randomly selected *Bufo* from each replicate of the 2 X *Bufo* treatment, were transferred to individual housing the same as in Experiment 1. Animals were maintained as before for 24 days. All survivors were euthanized and infection status of all experimental animals was again determined using qPCR^22^.

Experimental animals were coded as infected (1) or uninfected (0). In order to determine the significance of dose, host density and host diversity and interactions amongst these variables upon the probability of infection of *Bufo* we used generalised linear models with binomial errors, after removal of control animals from the dataset. Additionally, we investigated the effects of the different treatments upon the infection burden (mean GE) of infected *Bufo* using a generalised linear model with negative binomial errors. As for experiment 1, all statistical analyses were conducted in the software package R^28^.

## Results

### Experiment 1: Individual host species exposures and responses

**Figure 1:**
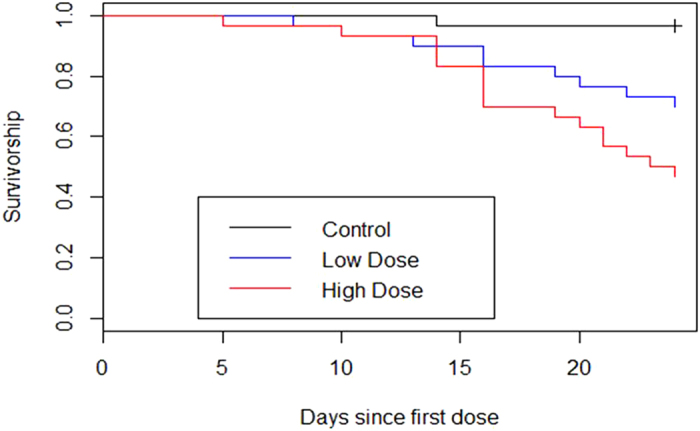
Kaplan-Meier survival plot for *Bufo bufo*, Experiment 1.

All but one *Rana* survived to the end of the experiment and none tested positive for infection via qPCR. We did not detect infection in any of the control and low dose treatment *Bufo*, including those that died before the end of the experiment. However, 80% of *Bufo* from the high dose treatment tested positive for *Bd*. Mortality was observed in all 3 *Bufo* treatment groups. In the minimum adequate model of CPH analysis both mass before exposure and dose were important predictors of survival in *Bufo*. Both high and low dose animals were significantly more likely to die than control animals, and within a dosage treatment lighter individuals were significantly more likely to die than heavier ones (Table 1, Fig. 1). Many of these animals died without any evidence of detectable infection, and infection status was not an important predictor of mortality in the minimum adequate model. The mass of individual *Bufo* before exposure to *Bd* did not significantly affect their post-exposure infection burden (z = 1.356, d.f. = 23, p = 0.175).

Exposure to *Bd* did not influence change in body mass of *Bufo* (ANOVA, F = 2.4699, d.f. = 2, p = 0.09), but we did detect an effect of dose on proportion change in body mass of *Rana*. *Post hoc* tests showed that *Rana* exposed to high doses gained significantly less weight, or lost weight, compared to animals from the control treatments (ANOVA, F = 3.3315, d.f. = 2, p = 0.04; Tukey’s HSD: control vs. low, p = 0.36; low vs. high, p = 0.44; control vs. high, p = 0.031).

### Experiment 2: Exposing single *Bufo*, cohoused *Bufo* and cohoused *Rana* and *Bufo*

**Figure 2:**
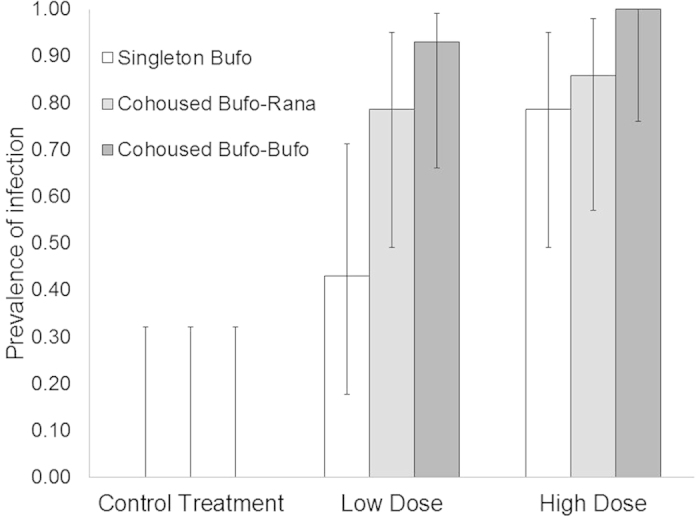
Prevalence of infection data of the nine experimental treatments in experiment two. GLM results show that density (z = 2.437, d.f. = 83, p = 0.0148) and dose (z = 2.269, d.f. = 83, p = 0.023) are important predictors of probability of infection in focal metamorphic *Bufo*, whereas diversity is not (z = 0.25, p = 0.25, Table 2). Error bars are 95% confidence intervals as calculated in QP30 software.

No animals died during the course of this experiment. Both dose (z = 2.269, d.f. = 83 p = 0.023) and host density (z = 2.437, d.f. = 83, p = 0.0148) were important predictors of an individual’s risk of infection. Increasing dose and increasing density of *Bufo* were both associated with an increased likelihood of infection (Fig. 2). When dose and host density were controlled for, host diversity was not a significant predictor of risk of infection (z = −1.143, d.f. = 83, p = 0.25, Table 2). None of the interaction terms included in the model-building process were found to be significant. The infection burden (mean GE) was not significantly affected by dose, host density or host diversity.

## Discussion

The ability of *Bd* to cause a wide variety of responses from exposed host species has been well described since the discovery of the parasite in 1998^29^,^30^,^31^. The continuum of host response ranges from those species that rarely become infected to those in which disease emergence may lead to mortality, and population decline. Although we have some understanding of how species or clades vary in response at the broadest end of this continuum, we still have relatively little knowledge of the implications of parasite exposure at the finer-scale. Our study highlights how two abundant European amphibian species vary greatly in their susceptibility to infection and, further, that they suffered varying costs associated not only with infection, but with parasite exposure. The negative consequences of avoiding infection that we observed suggest that the impacts of *Bd* upon host populations may be more subtle, yet further-reaching than previously considered.

We observed that susceptibility to infection varies between species, and that this variation was mirrored in species’ mortality rates. Our first experiment showed that toads were more susceptible to both infection and mortality as a result of exposure to *Bd*, and that both responses were dose-dependent (Table 1, Fig. 1). Further, the observed responses are consistent with *Bufo* response to *Bd* described in previous experiments^17^,^18^,^21^,^23^ in that infection with *Bd* at the end-point of the experiment was not always associated with mortality, and mass was a more important predictor of mortality than either infection with *Bd* or dose experienced (Table 1). In contrast, *Rana* appear to be resistant to *Bd* infection, which was not detected in this species even at high doses of *Bd* that resulted in near-complete infection of toads.

The most notable finding of our experiments was the negative effect of *Bd* exposure on hosts exhibiting no detectable infection. In the case of our susceptible host, *Bufo bufo*, we found that mortality often occurred at the low dose treatment without infection being detected. Given the apparent lack of infection in these individuals, the high levels of mortality observed, and the significant effect of start mass on survival, our results suggest that the costs of mounting an immune response to *Bd* are considerable, often resulting in death. Our results also suggest that the cost of mounting an immune response as a result of *Bd* exposure can lead not only to mortality, but also to significant sub-lethal effects. When our resistant host, *Rana temporaria*, were exposed to high doses, individuals exhibited significantly reduced growth and, in some cases, a reduction in mass, indicating that their effective resistance strategies may be costly. Such costs associated with immunity have been observed in other taxa^5^,^6^,^8^, with immune challenges leading to a reduction in parameters such as energy expended in reproduction, growth, and overall level of activity. How these individual-level changes scale-up to population level outcomes is not clear, but it seems likely that changes to the vital rates of species could result in population-level changes, even in species that do not become infected.

The presence of resistant species and the immune responses that they employ could have a beneficial effect on other species within an amphibian community. *Rana temporaria*, our resistant host, can mount both innate and adaptive immunological responses that are lethal for microparasites^32^,^33^,^34^ and produces antimicrobial peptides that are lethal specifically for *Bd*^35^. If resistant hosts are able to remove infectious zoospores from the environment without becoming infected these wasted transmission opportunities could lead to the occurrence of a dilution effect. Our first experiment showed that *Rana temporaria* does avoid infection, but the second experiment suggests that the presence of a more resistant host does not reduce the risk of infection of a co-housed susceptible host at the stage of initial infection. The data therefore provide no evidence of a dilution effect^1^ under these particular experimental conditions, which is contrary to a previous study of the existence of the dilution effect in the *Bd*-amphibian system^36^.

In the latter study, Searle *et al*. found that of the five indicators of disease risk measured in their experimental set-up (focal species infection prevalence; all-species infection prevalence; focal species infection severity; all-species infection severity; total infection), four showed evidence of a decrease with increasing species richness. In contrast, of the two measures of disease risk that we recorded (focal species infection prevalence and focal species infection severity), neither appeared to be affected by increases in diversity when accounting for host density. A number of factors may help to explain these apparently contradictory results. Our experiment aimed to look for evidence of the dilution effect in metamorphic animals at the initial stage of infection not at proliferation of infection as the transmission of infection occurred over multiple *Bd* lifecycles (hence our experimental animals were exposed to *Bd* for only 5 hours). In contrast, Searle *et al*. focussed upon larval amphibians over a much longer (35 day) period, with a greater overall density of animals. Combined, the effects of absolute density, age-class and duration of the experiment could help to explain the different results obtained. Additionally, within our experimental design we included only two treatments of diversity, which may have limited our ability to determine whether the dilution effect would be detected given a broader range of community structures. Similarly, for practical and ethical reasons, we were unable to replicate experiment 2 more than 14 times per treatment, which may have limited its statistical power and may therefore have led to a higher rate of type II errors than a larger experiment would have yielded. Further, larger, studies aiming to better understand transmission dynamics and the presence of the dilution effect under different scenarios of age-class, time-scale, density and diversity would be an interesting avenue for future research.

Increasing host density, even through the addition of a resistant host, did have a significant positive effect on the probability of infection of the susceptible host. When *Bufo* were exposed to low doses of *Bd*, cohousing with a second amphibian resulted in significantly increased probability of infection. Our experimental design eliminates the possibility that among-host transmission was a factor in infection dynamics, as experimental animals were exposed for only five hours, and zoospore encystment, maturation and sporulation occurs over a much longer time-span^37^. There are a number of possible mechanisms that could explain the positive relationship between infection and host density. Perhaps the most parsimonious explanation is that by increasing the number of hosts in the petri dish there is an increased level of host movement, and associated agitation of the water-body, which, in turn, would increase potential infectious contacts between zoospores and hosts. It is possible that experimental co-housing of toadlets caused stress that impairs immunity. We believe that this mechanism is highly unlikely as animals were co-housed at higher densities in single species groups for weeks before allocation to treatments. It seems improbable that co-housing at relatively low densities for five hours during exposures would have a more significant stress effect on immune function than the higher stocking density animals experienced before the start of the experiment. Another possible explanation, which has been predicted by theoretical models, is that in order to increase its R_0,_ a parasite may exhibit plastic infectivity to adapt to different scenarios of host availability and competition^38^,^39^; in the context of this experiment this would mean becoming more infectious as the number of available hosts increased. As infectivity is energetically costly, investment in the trait will be decided by a trade-off between host availability and zoospore lifespan, and it is therefore likely that there will be strong selection for adaptive plasticity in motile infectious stages. If *Bd* does exhibit adaptively plastic infectivity, it would add to the list of life history and behavioural traits that vary in response to different conditions^21^,^26^,^37^,^40^.

Our study provides insights that are important for the epidemiology of *Bd* and are of general importance for the understanding of wildlife disease dynamics. Experiment 1 illustrates how lethal effects associated with an infectious disease may not strictly correlate with infection status: *Bd* has the ability to affect individual host growth and survival without requiring detectable infection. We should therefore not assume that ascertaining the prevalence of infection is a reliable indicator of risk or impact of disease for all host species. Additionally, sub-lethal costs that impact amphibian growth will likely have an impact on the longer term survival and reproduction, at least when costs are imposed on juveniles^24^,^25^,^41^. As yet we have no understanding of how sub-lethal costs experienced by individual amphibians exposed to *Bd* translates to demographic responses, but they are likely to be influential^24^. Monitoring and quantifying the effects of *Bd* based purely on host infection-status and disease-related mortality rates may therefore lead to us underestimating the costs of *Bd* exposure and the impacts of this parasite upon host populations.

## Additional Information

**How to cite this article**: Bielby, J. *et al*. Host species vary in infection probability, sub-lethal effects, and costs of immune response when exposed to an amphibian parasite. *Sci. Rep*. **5**, 10828; doi: 10.1038/srep10828 (2015).

## Figures and Tables

**Table 1:** Cox proportional hazards model for survivorship in Experiment 1 in relation to dose and mass before exposure.

	Coefficient	SE	z	p
Low dose	2.653	1.060	2.504	0.0123*
High dose	3.463	1.037	3.34	<0.001**
Start mass	−0.092	20.08	−4.593	<0.001**

Represents minimum adequate model after removal of non-significant terms (infection status), d.f. = 3.

**Table 2:** Generalised linear model outputs for probability of infection in Experiment 2 as a function of infection dose, density of hosts, and diversity of hosts (d.f. = 83).

	Coefficient	SE	z	p
Density	2.7972	1.1476	2.437	0.0148*
Diversity	−1.3448	1.1771	−1.143	0.2532
Dose	1.4524	0.6401	2.269	0.0232*
